# Aminopeptidase B can bioconvert L-type amino acid transporter 1 (LAT1)-utilizing amide prodrugs in the brain

**DOI:** 10.3389/fphar.2022.1034964

**Published:** 2022-10-20

**Authors:** Agathe Hugele, Susanne Löffler, Belén Hernández Molina, Melina Guillon, Ahmed B. Montaser, Seppo Auriola, Kristiina M. Huttunen

**Affiliations:** School of Pharmacy, Faculty of Health Sciences, University of Eastern Finland, Kuopio, Finland

**Keywords:** amide, bioconversion, brain-targeted drug delivery, L-type amino acid transporter 1 (LAT1), peptidases, prodrug

## Abstract

A prodrug approach is a powerful method to temporarily change the physicochemical and thus, pharmacokinetic properties of drugs. However, in site-selective targeted prodrug delivery, tissue or cell-specific bioconverting enzyme is needed to be utilized to release the active parent drug at a particular location. Unfortunately, ubiquitously expressed enzymes, such as phosphatases and carboxylesterases are well used in phosphate and ester prodrug applications, but less is known about enzymes selectively expressed, e.g., in the brain and enzymes that can hydrolyze more stable prodrug bonds, such as amides and carbamates. In the present study, L-type amino acid transporter 1 (LAT1)-utilizing amide prodrugs bioconverting enzyme was identified by gradually exploring the environment and possible determinants, such as pH and metal ions, that affect amide prodrug hydrolysis. Based on inducement by cobalt ions and slightly elevated pH (8.5) as well as localization in plasma, liver, and particularly in the brain, aminopeptidase B was proposed to be responsible for the bioconversion of the majority of the studied amino acid amide prodrugs. However, this enzyme hydrolyzed only those prodrugs that contained an aromatic promoiety (L-Phe), while leaving the aliphatic promoeities (L-Lys) and the smallest prodrug (with L-Phe promoiety) intact. Moreover, the parent drugs’ structure (flexibility and the number of aromatic rings) largely affected the bioconversion rate. It was also noticed in this study, that there were species differences in the bioconversion rate by aminopeptidase B (rodents > human), although the *in vitro*–*in vivo* correlation of the studied prodrugs was relatively accurate.

## Introduction

Prodrug technology is a relatively old concept. The term “*pro-drug*” named by Adrien Albert was already introduced in 1958 (Albert, 1958). However, the technology itself was already used at the end of the 19th century. Antibacterial prodrug methenamine (or hexamine) and anti-inflammatory prodrug aspirin (acetylsalicylic acid) were launched by Schering and Bauer, respectively, in 1899 (Huttunen et al., 2011). Generally, prodrugs are inactive derivatives of pharmacologically active drugs and therefore they require chemical and enzymatic biotransformation after administration to release the active parent drug, such as salicylic acid in the case of aspirin. Thus, prodrugs are “masked” compounds, by which unfavorable physicochemical and/or pharmacokinetic properties of drugs can be temporarily altered. To date, the prodrug concept is well utilized; approximately 10% of all worldwide approved drugs can be considered prodrugs, and 12% of new small molecular entities approved by the U.S. Food and Drug Administration (FDA) in 2008–2017 were prodrugs (i.e., 30/250) (Rautio et al., 2018).

Nevertheless, not all prodrug applications have been successful. This is mainly due to the challenges in the translation phase from the rodent preclinical data to human clinical concepts (Huttunen et al., 2011; Hoppe et al., 2014). Species differences relating to bioconverting enzymes; their localizations, expression levels, and activities can be taken into account if the prodrug structures are designed to contain bonds that are biotransformed during the first-pass metabolism and not in specific tissues or cell types. Classical examples are phosphate and ester prodrugs that are hydrolyzed by ubiquitous phosphatases and carboxylesterases (CES), respectively (Satoh and Hosokawa, 2006; Di, 2019). Nevertheless, due to their uncontrolled bioconversion, ester prodrugs are quite rarely suitable for tissue-selective (targeted) prodrug delivery. Hence, other kinds of hydrolyzable prodrug bonds have been used, including amides, carbonates, carbamates, and thioesters, to increase the stability and prevent premature bioactivation and release of the parent drug during the first-pass metabolism (Ghosh and Brindisi, 2015; Simplício et al., 2008; Dansette et al., 2009; D'Souza and Topp, 2004). However, little is known of the enzymes hydrolyzing these prodrug bonds, particularly in specific organs, such as in the brain (Fukami and Yokoi, 2012; Prabha et al., 2013).

We have designed and developed numerous L-type amino acid transporter 1 (LAT1)-utilizing prodrugs from different parent drugs, including anti-parkinsonians, anti-inflammatories, anti-epileptic, antioxidants, and immunomodulatory drugs with the aim to improve brain drug delivery (Peura et al., 2013; Gynther et al., 2016; Huttunen et al., 2016; Puris et al., 2017; Thiele et al., 2018; Puris et al., 2019; Montaser et al., 2020). LAT1 is highly expressed in the brain; at the blood-brain barrier (BBB) as well as in the parenchymal cells (Boado et al., 1999; Huttunen et al., 2019), and it can carry not only large and neutral amino acids but also amino acid-mimicking (pro)drugs (Uchino et al., 2002). We have previously found that acetylcholinesterase (AChE), butyrylcholinesterase (BChE), and paraoxygenases (PONs) can bioconvert LAT1-utilizing ester prodrugs in addition to CES in the brain, peripheral tissues, and systemic circulation (Huttunen, 2018; Tampio et al., 2021). However, although ester prodrugs may have more effective uptake *via* LAT1 into the cells compared to their corresponding amide prodrugs (Huttunen et al., 2019; Venteicher et al., 2021), they may suffer from premature bioconversion during the first-pass metabolism and thus, the amide prodrugs may have advantages to deliver their parent drug, e.g., into the brain. Therefore, the aim of the present study was to identify the possible enzymes responsible for bioconversion of LAT1-utilizing amide prodrugs, particularly in the brain. We have reported that many LAT1-utilizing amide prodrugs are very stable when studied *in vitro* in tissue homogenates, but they have released their parent drugs *in vivo* in mice, and curiously some of them selectively in the brain (Table 1) (Puris et al., 2017; Montaser et al., 2019; Puris et al., 2019). Therefore, it has been hypothesized that the used *in vitro* assays may lack some essential co-factors required for the optimal function of the bioconverting enzymes.

**TABLE 1:** The studied compounds; chemical structures (the promoiety highlighted with red color) and the extent of bioconversion *in vivo* in mice, accompanied by the literature references reporting the synthesis of the compounds and their *in vivo* bioconversion. The parent drug of prodrugs **1**–**4** is ketoprofen, prodrug **5** is ferulic acid, prodrug **6** salicylic acid, prodrug **7** flurbiprofen, prodrug **8** ibuprofen, and prodrug **9** naproxen.

	Prodrug structure	*In vivo* bioconversion	References
1	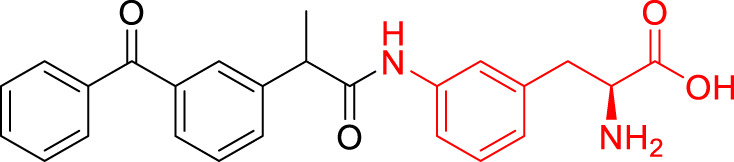	∼50% bioconversion in brain	Puris et al. (2017)
		<1% bioconversion in liver	
		∼50% bioconversion in plasma	
2	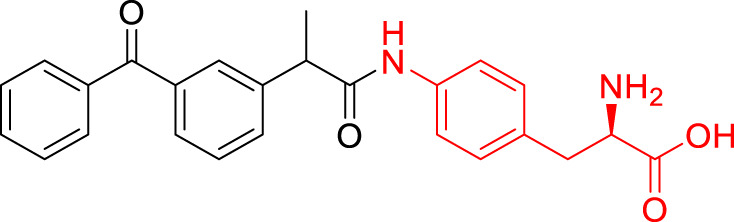	∼50% bioconversion in brain	Puris et al. (2017)
		<1% bioconversion in liver	
		∼30% bioconversion in plasma	
3	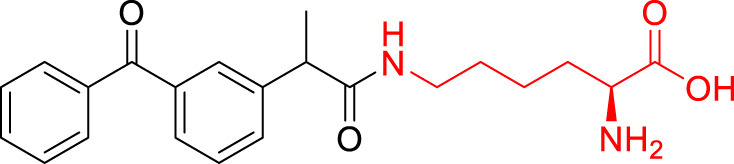	No bioconversion in brain	Puris et al. (2017)
		<1% bioconversion in liver	
		<1% bioconversion in plasma	
4	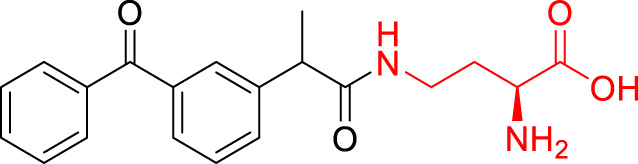	No bioconversion in brain	Puris et al. (2017)
		<1% bioconversion in liver	
		<10% bioconversion in plasma	
5	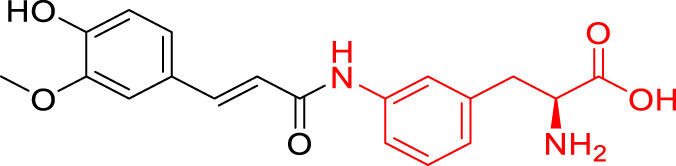	∼5% bioconversion in brain	Puris et al. (2019), Tampio et al. (2022)
		<1% bioconversion in liver	
		∼80% bioconversion in plasma	
6	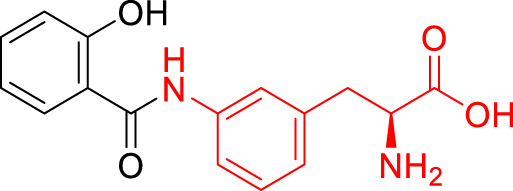	∼80% bioconversion in brain, no bioconversion in liver	Montaser et al. (2020)
		<1% bioconversion in plasma	
7	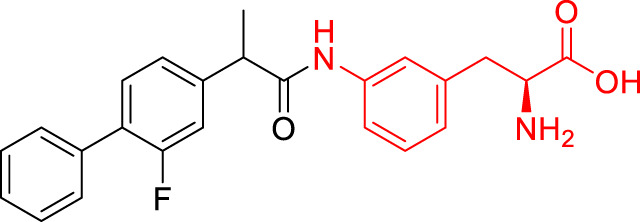	No bioconversion in brain	Montaser et al. (2020)
		No bioconversion in liver	
		∼20% bioconversion in plasma	
8	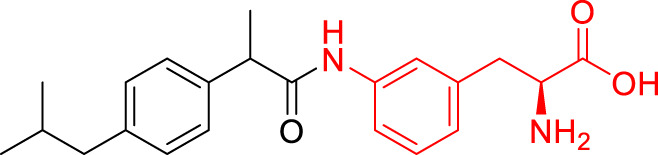	No bioconversion in brain	Montaser et al. (2020)
		No bioconversion in liver	
		∼20% bioconversion in plasma	
9	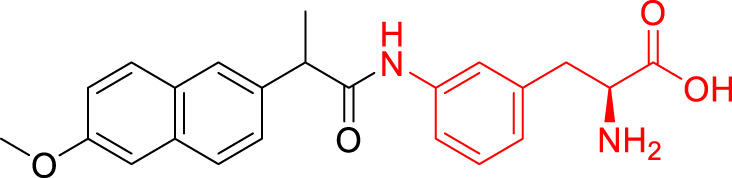	∼50% bioconversion in brain, no bioconversion in liver	Montaser et al. (2020)
		∼30% bioconversion in plasma	

Since the herein studied compounds contain amide prodrug bonds, it was expected that the possible bioconverting enzyme belongs to a group of hydrolyzing enzymes acting on peptide bonds (enzyme class 3.4), namely peptidases. Within the peptidase class, there are many different subgroups and enzymes that are also sensitive to the surrounding pH. For example, cysteine proteases (EC 3.4.22) and aspartic proteases (EC 3.4.23) display their best activity at low pH (ca. 2–6), while serine proteases (EC 3.4.21) and metalloproteases (EC 3.4.24) are regarded as neutral enzymes (Fasciglione et al., 2000; Verma et al., 2016; Hofer et al., 2020). Many peptidases require also a coordination metal ion, such as zinc (Zn^2+^), for their activity and therefore they are called metalloproteases. As LAT1 carries the prodrugs into the cells, it was expected that the possible bioconverting enzyme is localized in the cytosol or intracellular membranes, such as endoplasmic reticulum (ER) rather than outside of the cells (secreted). Therefore, in this study, it was evaluated how different metal ions and pH-changes affect the bioconversion of the prepared LAT1-utilizing amide prodrugs *in vitro* in different biological media, including human plasma, and rodent liver and brain subcellular S9 fractions, which are rich in hydrolytic enzymes. The bioconversion rates of the LAT1-utilizing prodrugs were also tested in astrocytic and microglial homogenates and with the suspected recombinant enzyme. Lastly, the expression levels of the candidate prodrug bioconverting enzyme were evaluated from the mouse brain, liver, and plasma (the tissues and fluid that were evaluated in the pharmacokinetic studies previously; Table 1). Thus, this study sheds light on how successful brain-targeted prodrugs can be designed in the future and discusses possible benefits and obstacles that this kind of transporter-utilizing brain-targeted prodrug approach may have.

## Materials and methods

### Chemicals

All reagents and solvents used in analytical studies were commercial and high purity of analytical grade or ultra-gradient HPLC-grade purchased from MilliporeSigma (St. Louis, MO, United States), ThermoFisher Scientific (Waltham, MA, United States), J. T. Baker (Deventer, Netherlands), Riedel-de Haën (Seelze, Germany), Promega Biotech AB (Nacka, Sweden), or EuroClone S. p.A. (Pero, Italy). Water was purified using a Milli-Q Gradient system (Millipore, Milford, MA, United States). Synthesis, structural characterization (^1^H NMR, ^13^C NMR, LC-MS), and over 95% purity (elemental analysis) of the studied LAT1-utilizing prodrugs have been reported earlier (Table 1).

### Biological material

Rat and mouse liver and brain S9 fractions were prepared by centrifuging liver and brain homogenates at 9,000 × *g* for 20 min at 4°C and collecting the supernatant. The liver and brain homogenates were prepared by homogenizing freshly collected rat or mouse liver or brain with 50 mM Tris-buffered saline (TBS) (pH 7.4) (1:4 w/v). Protein concentrations of all fractions were determined by Bio-Rad Protein Assay, based on Bradford dye-binding method (EnVision, Perkin Elmer, Waltham, MA, United States). The control tissues were obtained as a part of other animal experiments, which were made in compliance with the European Commission Directives 2010/63/EU and 86/609, and approved by the Institutional Animal Care and Use Committee of the University of Eastern Finland (Animal Usage Plan number ESAVI/3347/04.10.07/2015). All biological material was stored at −80°C until used.

The pooled mouse and human plasma were obtained from control animals or healthy human donors by collecting whole blood aseptically and centrifuging at 12,000 × *g* for 10 min. Human recombinant aminopeptidase B (1 mg/ml protein) produced in *E. Coli*, was purchased from ProSpec-Tany TechnoGene Ltd. (Ness Ziona, Israel).

Primary astrocytes from the cortex and hippocampi were isolated from 2-days-old mice as previously described (Jankowsky et al., 2004; Pihlaja et al., 2008). The astrocytes (passages 16–26) and immortalized microglia (BV2; passages of 13–25) were cultured in Dulbecco’s Modified Eagle Medium F-12 Nutrient Mixture (DMEM/F2) and RPMI-1640 medium, respectively, both supplemented with l-glutamine (2 mM), heat-inactivated fetal bovine serum (10%), penicillin (50 U/ml) and streptomycin (50 μg/ml). Astrocyte or BV2 microglia homogenates were prepared by sonicating the cell suspensions (0.5–1.0 × 10^6^ cells/ml) in TBS. The protein content was determined as described above (Bio-Rad Protein Assay) and the cell homogenates were used as such.

### Ultra-high-performance liquid chromatography analyses

The amount of the studied prodrugs and their parent drugs were determined by the ultra-high-performance liquid chromatography (UPLC) system (Agilent Technologies 1290 Infinity II system; Agilent Technologies Inc., Wilmington, DE, United States), which consisted of a high-speed pump, a multi-sampler, a multi-column thermostat (MCT), and a diode array detector (DAD). The chromatographic separations were achieved on an Agilent ZORBAX Eclipse Plus C18 analytical column (2.1 × 50 mm, 1.8 µm) (Agilent Technologies Inc., Wilmington, DE, United States) by using isocratic elution of water (A) containing 0.1% formic acid (pH ca. 3.0) and acetonitrile (B) containing 0.1% formic acid at the flow rate of 1.0 ml/min at room temperature. Prodrugs **1–4** and **7**, ketoprofen and flurbiprofen were recorded at 250 nm, prodrug **5** and ferulic acic at 325 nm, prodrugs **6** and **9**, salicylic acid and naproxen at 230 nm, and prodrug **8** and ibuprofen at 226 nm. The retention times for prodrugs **1–4** were between the range of 1.44–1.91 min and for ketoprofen 3.29 min with an elution ratio of 72:28 (v/v), for prodrug **5** 2.33 min and for ferulic acid 1.48 min with an elution ratio of 88:12 (v/v), for prodrug **6** 0.95 min and for salicylic acid 1.47 min with an elution ratio of 85:15 (v/v), for prodrug **7** 1.26 min and for flurbiprofen 4.04 min with an elution ratio of 65:35 (v/v), for prodrug **8** 1.83 min and ibuprofen 2.59 min with an elution ratio of 60:40 (v/v), for prodrug **9** 1.42 min and naproxen 2.74 min with an elution ratio of 70:30 (v/v). The UPLC methods were accurate (100% ± 10%), precise (RSD <10%), and specific (no interfering peaks were observed) over the range of 0.2–20 μM. The lowest limit of detection varied from 0.02 to 0.1 μM with prodrugs and their parent drugs.

### Bioconversion of prodrugs

The rates of bioactivation of prodrugs in rat, mouse, and human liver S9 fractions, in rat and mouse brain S9 fractions or cell homogenates were determined at 37°C. The incubation mixtures were prepared by mixing liver or brain S9 fraction, or cell homogenate (final protein concentration 1.0 mg/ml) with TBS buffer (pH 7.4), 10 mM metal ion solution (2 mM; MgCl_2_, CaCl_2_, CuCl_2_, NiCl_2_, ZnCl_2_, or CoCl_2_) and 10 mM prodrug stock solution in DMSO (the final concentrations of prodrugs were 100 µM and the DMSO concentration 2%). The mixture was then incubated for 5 h and the samples (100 µl) were withdrawn at appropriate intervals. Relatively high concentrations of the studied compounds were used to ensure reliable quantification after sample preparation. The proteins in the samples were precipitated with ice-cold acetonitrile (100 µl) and the samples were centrifuged for 5 min at 12,000 × *g* at room temperature. The supernatants were collected and analyzed by the UPLC method described above. In blank reactions (pH 6.5, 7.4, and 8.5), the metal ion solution was replaced with the same volume of buffer, in inhibition studies (pH 7.4) 10 mM bestatin (final concentration 100 µM) was added to the incubation mixture, and in the reaction with aminopeptidase B (pH 8.5), S9 subcellular fraction was replaced with pure recombinant enzyme (100 μg/ml protein). The pseudo-first-order half-lives (*t*_*½*_) for the rates of bioconversion of the prodrugs were calculated from the slope of the linear portion of the plotted logarithm of remaining prodrug concentration versus time, which was noted to be linear over the studied period (5 h), implying that the enzyme was fully functional in the studied media during the assays.

### Non-targeted (global) proteomics of the used biological material

The BV2 cell lysates were denatured, reduced, and carboxymethylated prior to the digestion with LysC and TPCK-trypsin, as described previously (Montaser et al., 2020). Then, a total amount of 50 μg protein was digested by LysC (1:100, w/w) and 0.05% ProteaseMax for 3 h followed by trypsin digestion (1:100, w/w) for 18 h at 37°C. The tryptic digestion was quenched with formic acid. Subsequently, the samples were centrifuged at 18,000 × *g* for 5 min at 4°C, and the supernatants were transferred into UPLC vials for the analysis.

The tryptic peptides (10 µg) were analyzed by UPLC (Vanquish Flex, Thermo Scientific, Bremen, Germany) coupled with a high-resolution mass spectrometer (MS) (Orbitrap Q Exactive Classic, Thermo Scientific, Bremen, Germany) following the full scan and data-independent acquisition mode (DIA). The injected peptides were first separated by reversed-phase chromatography composed of an Agilent AdvanceBio Peptide Map 2.1 mm × 250 mm, 2.7 μm column (Agilent Technologies, Santa Clara, CA, United States) and eluents water (eluent A) and acetonitrile (eluent B) acidified with 0.1% (v/v) formic acid. The injection volume was 20 µl and the flow rate was 0.3 ml/min over a direct gradient of 2% B for 80 min followed by a washing step of 80% B for 7 min before equilibrating the gradient back to 2% B for 3 min. An active gradient of 0–80 min was analyzed in the positive polarity by Full MS–SIM mode (Resolution: 35000, AGC target: 3e6, maximum injection time: 60 ms, and scan range: 385–1015 m/z) and DIA mode (Resolution: 17500, AGC target: 2e6, maximum injection time: 60 ms, loop count: 25, and isolation window: 24 m/z).

The raw data was processed by DIA-NN software (version 1.8) using the library-free DIA analysis mode (Demichev et al., 2020). The MS/MS spectra and retention time were predicted by the DIA-NN algorithm using the Uniprot reference proteome database for *Mus musculus* (UP000000589_10090; version from 2021-06-16; 22,001 protein sequences). The predicted MS library was used to search the raw data with 1% precursor and protein group (PG) false detection rate (FDR) thresholds and at least one proteotypic peptide with a length of 7–30 amino acids. The resulting MaxLFQ normalized intensities were used for data evaluation (Cox et al., 2014).

### Data analysis

All the studies were carried out as three replicates and presented as mean ± SD, (*n* = 3). Statistical analyses were performed using GraphPad Prism v. 5.03 software (GraphPad Software, San Diego, CA, United States). Statistical differences between the groups were tested using one-way ANOVA, followed by a two-tailed Tukey’s multiple comparison test and presented as mean ± SD, with statistically significant differences denoted by *p < 0.05, **p < 0.01, ***p < 0.001.

## Results

### Bioconversion of L-type amino acid transporter 1-utilizing aromatic amide prodrugs is pH- and cobalt-dependent

**FIGURE 1:**
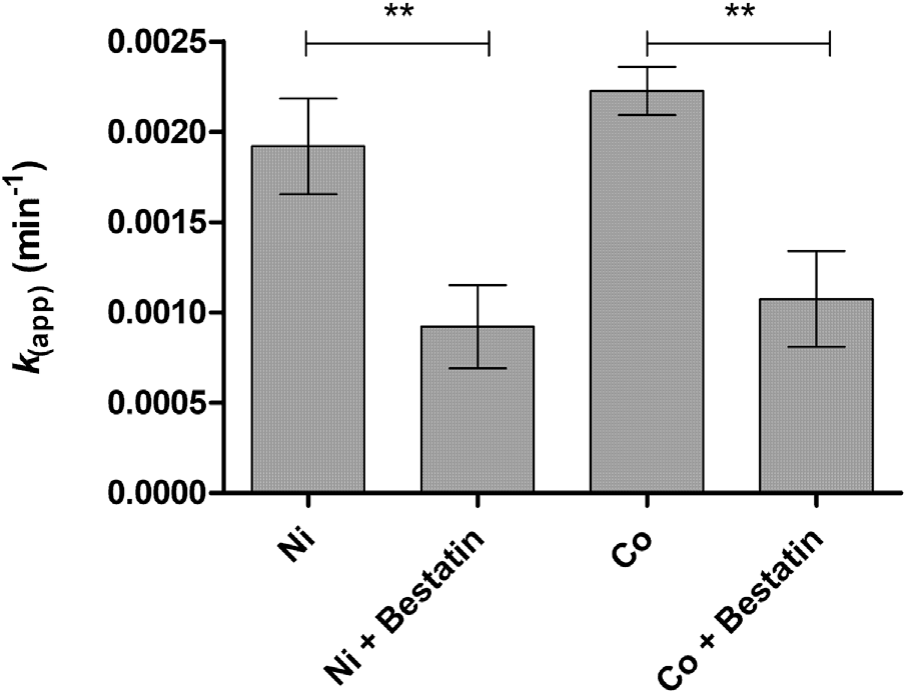
Bioconversion rate constants [*k* (*app*)] of prodrug **1** (100 µM) in rat liver S9 subcellular fraction in the presence of 2 mM cobalt, with and without non-specific metalloprotease inhibitor, bestatin (100 µM). Data are presented as mean ± SD (*n* = 3) and an asterisk denotes a statistically significant diﬀerence from the respective control (**p < 0.01, one-way ANOVA, followed by Tukey’s multiple comparison test).

To explore the possible LAT1-utilizing amide prodrug bond containing compounds bioconverting enzymes and understand the bioactivation mechanisms in more detail, the compounds’ enzymatic stability was studied in human plasma and rat liver and brain S9 subcellular fractions by altering the studied conditions. First, prodrug **1** was predisposed to slightly acidic (pH 6.5) and slightly basic (pH 8.5) conditions at +37°C, which did not activate the bioconverting enzyme(s) as such (*data not shown*). Then, divalent coordination metals, including magnesium (Mg^2+^), calcium (Ca^2+^), copper (Cu^2+^), nickel (Ni^2+^), zinc (Zn^2+^), and cobalt (Co^2+^), were added in the biological media (pH 7.4). From the added metal ions, magnesium, calcium, and copper did not affect the bioconversion, while nickel was able to induce enzymatic hydrolysis in media of rodent origin to some extent, t_½_ being in rat liver and brain S9 subcellular fractions 6 h or over, respectively (Table 2). Surprisingly, zinc did not have major effects; only minor bioconversion was seen in rat liver S9 subcellular fraction (7% ± 2% of the prodrug was bioconverted during the first 3 h). However, cobalt was able to activate bioconverting enzymes in all studied media, increasing the bioconversion rate most effectively in human plasma (t_½_ ca. 65 min), followed by rat brain S9 fraction (t_½_ ca.129 min) and rat liver S9 fraction (t_½_ ca. 312 min). Decreasing pH in rat liver S9 fraction below 7.4 in the presence of cobalt decelerated the bioconversion rate to zero, while increasing pH to 8.5 accelerated the bioconversion rate, half-life dropping from 312 ± 19 min to 178 ± 19 min (Table 2). Adding a non-specific metalloprotease inhibitor, bestatin (100 µM) to nickel- and cobalt-catalyzed reactions, the bioconversion rate (*k*_*app*_) dropped approximately by half (Figure 1). As the bioconversion in these circumstances was relatively slow, the yield of the parent drug (ketoprofen in the case of prodrug **1**) was not quantitative.

**TABLE 2:** Bioconversion of prodrug **1** (100 µM) in human plasma as well as in rat liver and brain S9 subcellular fractions in the absence and presence of 2 mM metal ions at pH 7.4 (+37°C). The results are presented as half-lives (mean ± SD, *n* = 3).

pH 7.4 (unless otherwise stated)	Human plasma (min)	Rat liver S9 (min)	Rat brain S9 (min)
Blank	N.B.	N.B.	N.B.
Mg^2+^	N.B.	N.B.	N.B.
Ca^2+^	N.B.	N.B.	N.B.
Cu^2+^	N.B.	N.B.	N.B.
Ni^2+^	N.B.	366 ± 45	>360
Zn^2+^	N.B.	Minor	N.B.
Co^2+^	65 ± 2	312 ± 19	129 ± 7
Co^2+^, pH 6.5	—	N.B.	—
Co^2+^, pH 8.5	—	178 ± 19	—

N.B. denotes “*no bioconversion*”.

The dash (—) abbreviates that the experiment was not carried out in these conditions.

Thus, since no bioconversion with prodrug **1** was observed in human plasma, rat liver, or brain S9 subcellular fractions without metal ions, the possible bioconverting enzyme class narrowed to metalloproteinases (EC 3.4.21). The sensitivity to slightly alkaline conditions also limited the number of candidate bioconverting enzymes for LAT1-utilizing amide prodrugs. However, at this point, it was important to notice that there is also another enzyme class, aminopeptidases (EC 3.4.11) that are also metalloenzymes, but they differ from the metalloproteinases (EC 3.4.21) in their activities; activation mechanisms and active site of the enzymes (Mucha et al., 2010).

Curiously, most of the other LAT1-utilizing amide prodrugs behaved similarly, adding cobalt into the studied media activated the bioconversion (prodrugs **2**, **5**, **7**, **8**, and **9**) (Table 3). When the cobalt was replaced with other metal ions or metalloprotease inhibitor bestatin was added to the media, the bioconversion rate decelerated remarkably, if occurred at all. Curiously, the bioconversion in human-derived media the studied prodrugs behaved differently; in plasma the bioconversion was faster compared to liver S9 subcellular fraction (Table 3). Prodrugs having aliphatic amino acid promoiety (L-Lys with compounds **3** and **4**), were not bioconverted despite the addition of the activating metal ion (Table 3), indicating that these prodrugs were not as good substrates for the bioconverting enzyme as the other prodrugs having aromatic amino acid promoiety (L-Phe with prodrugs **1**, **2**, **5**, **7**, **8**, **9**). The only exception seemed to be compound **6**, which was resistant to the bioconversion in the presence of metal ions, implying that it may be bioconverted by other enzymes than the rest of the aromatic LAT1-utilizing amide. Moreover, compound **6** is structurally much smaller and more water-soluble compared to the other larger and lipophilic LAT1-utilizing compounds, which most likely affects the interactions between the compounds and the protein, required for the hydrolysis of the prodrugs.

**TABLE 3:** Bioconversion of studied compounds (100 µM) in rat liver and brain S9 subcellular fractions as well as in human plasma and liver S9 subcellular fraction (pH 7.4) and with human recombinant aminopeptidase B (pH 8.5) in the presence of 2 mM Co^2+^ (+37°C). The bioconversion rates were also inhibited (*inh.*) with metalloprotease inhibitor bestatin. All the results are presented as half-lives (mean ± SD, *n* = 3).

	Rat liver S9 (min)	Rat brain S9 (min)	Human plasma (min)	Human liver S9 (min)	Human aminopeptidase B (min)
1	312 ± 19	129 ± 7	65 ± 2	N.B.	24 ± 1 (60%, in 30 min)
*Inh.*	*—*	*>360*	*138 ± 18*	*—*	
2	245 ± 77	N.B.	103 ± 11	N.B.	20 ± 2 (40% in 20 min)
*Inh.*	652 ± 87***	*—*	*123 ± 26*	*—*	
3	N.B.	N.B.	>360	N.B.	N.B.
4	N.B.	N.B.	N.B.	N.B.	N.B.
5	>360	>360	37 ± 5	N.B.	216 ± 15 (55% in 4 h)
*Inh.*	*—*	*—*	75 ± 22***	*—*	
6	N.B.	N.B.	N.B.	N.B.	N.B.
7	141 ± 8	>360	>360	>360	35 ± 2 (50% in 40 min)
*Inh.*	185 ± 14**	*—*	*—*	*—*	
8	216 ± 15	>360	>360	N.B.	208 ± 28 (60% in 4 h)
*Inh.*	303 ± 30*	*—*	*—*	*—*	
9	>360	N.B.	N.B.	N.B.	58 ± 5 (30% in 30 min)
*Inh.*	*—*	*—*	*—*	*—*	

N.B. denotes “*no bioconversion*” and an asterisk statistically significant diﬀerence from the respective control

***p < 0.001, **p < 0.01, *p < 0.05 one-way ANOVA, followed by Tukey’s multiple comparison test.

The dash (*—*) abbreviates that the experiment was not carried out in these conditions.

### Bioconversion of L-type amino acid transporter 1-utilizing aromatic amide prodrugs is mediated *via* aminopeptidase B

Thus, we concluded that the possible LAT1-utilizing bioconverting enzyme for most of the prodrugs could be aminopeptidase B (EC 3.4.11.6, RNPEP, APB, arginyl aminopeptidase), based on the utilization of cobalt and the pH-dependency as well as its existence in the brain (Ramírez et al., 1990; Fukasawa et al., 1996; Foulon et al., 1999; Ramírez-Expósito et al., 2001; Fukasawa et al., 2011). Therefore, the bioconversion of the prodrugs was also determined with the human recombinant aminopeptidase B in the presence of cobalt at pH 8.5. As seen in table 3, this recombinant enzyme indeed bioconverted the same aromatic amino acid prodrugs (**1**, **2**, **5**, **7**, **8**, **9**) that were prone to the cobalt-mediated hydrolysis in the human plasma, rat liver, or brain S9 subcellular fractions, while the highly stable aliphatic amino acid prodrugs (**3** and **4**) and the exceptional prodrug **6** were not bioconverted by this enzyme. There was also a wide variation in the bioconversion rate, with most prodrugs having half-lives less than 1 h (compounds **1**, **2**, **7**, and **9**). However, there were also slowly metabolized compounds; prodrugs **5** and **8** had half-lives over 3 h. Curiously, these prodrugs (**5** and **8**) have only one aromatic ring in their parent drug structure, while the more rapidly metabolized prodrugs (**1**, **2**, **7**, and **9**) have two aromatic rings in addition to the third aromatic ring of the amino acid promoiety. Thus, it can be concluded that the structure of the overall prodrug defines the bioconversion rate of these amino acid amide prodrugs.

To cross-validate our conclusions with another rodent species, by which the pharmacokinetic studies were carried out previously (Table 1), and to attain reliable *in vitro-in vivo* correlation between the achieved results, the bioconversion rate was also studied in mouse serum and liver/brain S9 subcellular fractions as well as immortalized mouse microglia (BV2 cells) and astrocytes (Puris et al., 2017; Puris et al., 2019; Montaser et al., 2020). The bioconversion rate in a biological media of mouse origin followed the *in vivo* pharmacokinetic study mouse (Tables 1, 4), where aliphatic amino acid prodrugs did not release their parent drug at all (compounds **3** and **4**), particularly in the mouse brain (Puris et al., 2017). Contrarily, the aromatic amino acid prodrugs (compounds **1**, **2**, **5**, **7**, **8**, **9**) released their parent drug in all mouse media as well as *in vivo* (excluding compound **6**) (Puris et al., 2017; Puris et al., 2019; Montaser et al., 2020), which is also in accordance with the results obtained with enzymes derived from rat and human media (Table 3). Only with prodrugs **7** and **8**, no released parent drug was observed *in vivo* in the brain, although *in vitro,* they were hydrolyzed and released their parent drugs (flurbiprofen and ibuprofen) both in rat and mouse brain subcellular fractions (Tables 3, 4). Both these parent drugs can undergo further metabolism (Karlsson and Fowler, 2014), and therefore, it is highly likely that in the pharmacokinetic study, in which the metabolites were not followed, the release of these parent drugs cannot be truly estimated. Moreover, prodrugs **7** and **8** were also bioconverted with the human recombinant aminopeptidase B, which supports the conclusion, that these compounds were also aminopeptidase B substrates. With prodrug **6** no correlation was seen between *in vitro* and *in vivo* results, indicating that some other enzyme rather than aminopeptidase B is responsible for the hydrolysis and release of its parent drug (salicylic acid) *in vivo*. This compound also had the highest bioconversion extent in the mouse brain (ca. 80% of the prodrug was bioconverted), and lowest bioconversion extent in mouse plasma and liver (less than 1% and no bioconversion, respectively) (Montaser et al., 2020), which makes the possible bioconverting enzyme(s) participating in the hydrolysis of prodrug **6** very interesting, particularly from the brain-targeted drug delivery point of view.

**TABLE 4:** Bioconversion of studied prodrugs (100 µM) in mouse serum (50%) as well as mouse liver and brain S9 subcellular fractions in the presence of 2 mM Co^2+^ at pH 7.4 (+37°C). Results are presented as half-lives (mean ± SD, *n* = 3) and N.B. denotes “*no bioconversion*”.

	Mouse serum (50%) (min)	Mouse liver S9 fraction (min)	Mouse brain S9 fraction (min)	Mouse microglia (min)	Mouse astrocytes (min)
1	323 ± 19	>360	>360	>360	394 ± 31
2	>360	>360	>360	>360	>360
3	>360	N.B.	N.B.	N.B.	N.B.
4	>360	N.B.	N.B.	N.B.	N.B.
5	133 ± 3	141 ± 19	N.B.	>360	>360
6	N.B.	N.B.	N.B.	N.B.	N.B.
7	226 ± 8	238 ± 17	318 ± 80	119 ± 7	144 ± 14
8	211 ± 18	267 ± 24	266 ± 11	>360	>360
9	>360	>360	>360	N.B.	N.B.

### Aminopeptidase B can be a brain-selective prodrug bioconveting enzyme

Curiously, there were only minor differences in the bioconversion rate between mouse serum, liver S9 fraction, and brain S9 fraction (Table 4). In turn, the bioconversion was to some extent faster in the cellular homogenates, although the total protein amount was the same in all bioconversion studies. Microglia and astrocytes homogenates have most likely, a higher content of the responsible enzyme in relation to other proteins, and compared to the S9 subcellular fractions prepared from tissue homogenates. As can be seen from Tables 3, 4, species differences were also noticed between humans and rodents as well as among the rodents (mice vs. rats). In addition, there were also differences among the prodrugs having aromatic promoiety. The most flexible parent drugs 5, 7, and 8 were readily hydrolyzed followed by structurally more rigid compounds (1 > 2 > 9). Thus, in addition to the number of aromatic rings, the flexibility of the parent drug can have a major impact on the bioconversion rate of these amino acid amide prodrugs.

**FIGURE 2:**
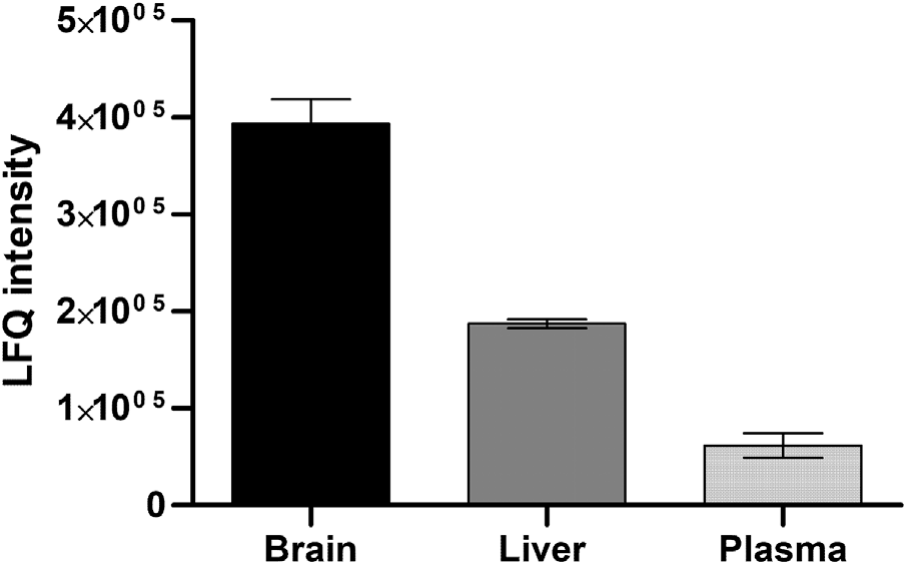
Relative protein expression of aminopeptidase B measured by non-targeted global proteomic approach from mouse brain and liver tissues and plasma (mean ± SD, *n* = 3).

**FIGURE 3:**
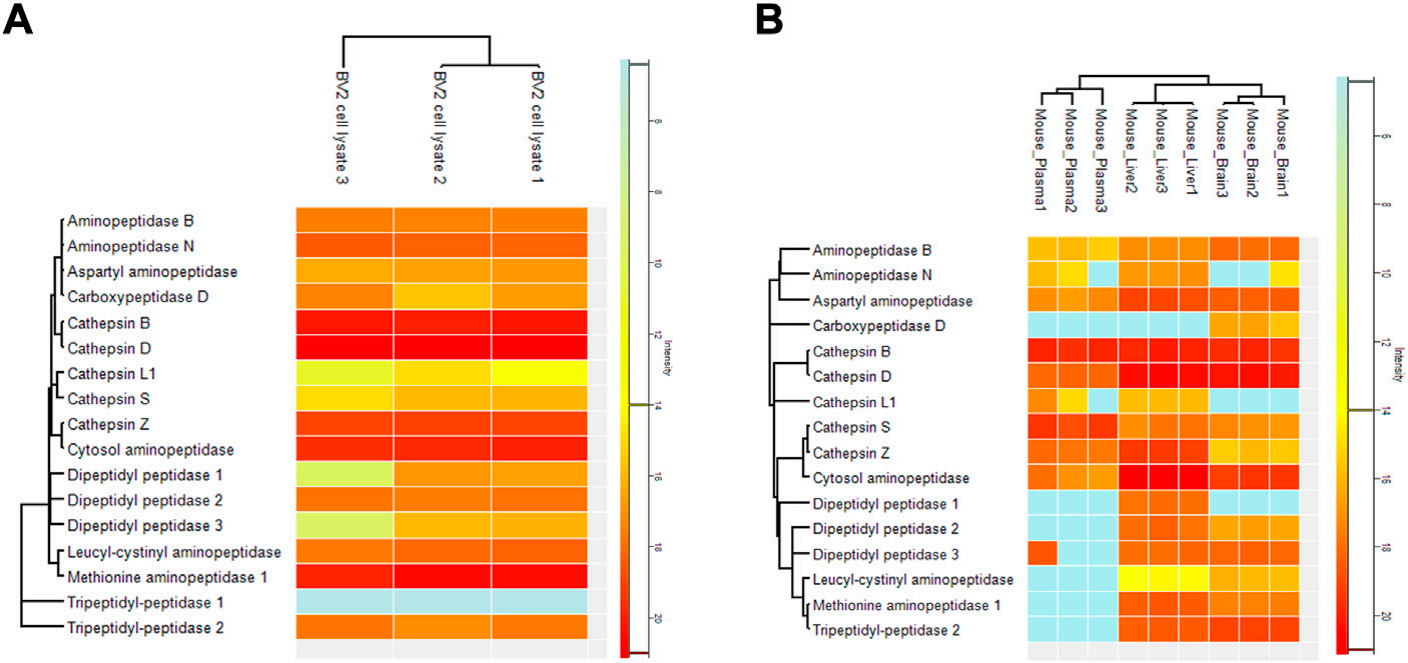
Relative protein expression of selected enzymes measured by non-targeted global proteomic approach from **(A)** cell homogenate of mouse BV2 microglia, and **(B)** mouse brain and liver tissues as well as from plasma. The results are presented as heatmap with red illustrating the highest intensity and blue the lowest intensity.

To confirm that the cobalt and pH-dependent enzyme (aminopeptidase B) that was identified in the *in vitro* studies, was also responsible for the prodrug bioconversion seen in the pharmacokinetic studies with mice, a global proteomic study was carried out for the mouse plasma as well as tissue and cell homogenates. By this non-targeted method, only fold differences between the tissues can be compared and it cannot be considered an absolute quantification. However, in this experiment, several specific peptides related to aminopeptidase B were recognized, which increases the accuracy of the enzyme identification. The highest expression of aminopeptidase B was found in the mouse brain, followed by liver and plasma (Figure 2). Since LAT1 is also expressed in the brain parenchymal cells (Huttunen et al., 2019), and thus it carries its substrates into the brain parenchymal cells (neurons, astrocytes, and microglia), aminopeptidase B expression in mouse BV2 microglia was also analyzed. Curiously, it was found that mouse immortalized microglia expressed several other potential prodrug bioconverting enzymes, particularly other aminopeptidases, including also di- and tripeptyl peptidases, as well as cathepsins, in addition to aminopeptidase B (Figure 3).

## Discussion

Although the prodrug concept is a relatively old and widely utilized approach in drug discovery and development, less attention has been paid to site-selective bioconversion of more stable prodrugs, such as amides, and their bioconverting enzymes (Simplício et al., 2008; Eichenbaum et al., 2012; Gynther et al., 2016; Huttunen, 2018; Di, 2019; Tampio et al., 2021). Therefore, the *in vitro*–*in vivo* correlations have been less accurate and the success of site-selectively released prodrugs in the clinical context has been more or less sporadic. However, as long as the pharmacological responses have been improved in comparison to their parent drugs and the clinical outcomes have been successful, the need of clarifying these bioconverting enzymes has been, unfortunately, less warranted.

Brain drug delivery is a very challenging part of the central nervous system (CNS)-drug development, due to the highly restricting BBB that protects the brain from *xenobiotics*, while retaining homeostasis of many endogenous compounds (Pardridge, 2012; Pardridge, 2022). Therefore, taking advantage of specific delivery routes of endogenous compounds, such as transporters selectively expressed at the BBB, can be one way to improve brain drug delivery (Huttunen et al., 2022). To mimic endogenous compounds without compromising the potency of drug candidate towards the final target, the prodrug approach can be used. However, there is still a lack of knowledge of brain-specific enzymes. It is highly important in the targeted prodrug approach to avoid premature hydrolysis *e.g.*, during the first-pass metabolism, and to attain efficient site-selective bioconversion. Moreover, greater controlled release prodrugs could be designed if the bioconverting enzymes were recognized and more information about their binding pockets and the catalytic mechanisms were available that could be taken into account in the structural prodrug design.

In the present study, it was identified that aminopeptidase B (3.4.11.6, RNPEP, APB, arginyl aminopeptidase) is one of the key enzymes hydrolyzing LAT1-utilizing amide prodrugs, and particularly in the rodent brain tissue and cell homogenates (Table 3, 4). It was activated in the presence of cobalt ions and slightly elevated pH (8.5) and inhibited by non-specific protease inhibitor bestatin (Tables 2, 3; Figure 1). The enzyme was also localized and active in human and mouse plasma, and it was more effective in rat and mouse liver subcellular fractions compared to one of humans. Aminopeptidase B was has been identified in human liver and plasma already in 1968 and 1976, respectively (Mäkinen, 1968; Mäkinen and Virtanen, 1976), and it has been found as secreted extracellular isoform as well as intracellularly in Golgi apparatus (Balogh et al., 1998). Although it may have a low tissue specificity or low brain regional specificity, as stated by the human protein atlas (www.proteinatlas.org, read 1.7.2022), more attention should be paid to its quantitative protein expression and functional capacity in different tissues. In the present study, the semi-quantitative expression of aminopeptidase B revealed that mouse brain has 2-times higher levels of this enzyme compared to the amounts in mouse liver, and 4-times higher amounts compared to mouse plasma (Figure 2). Therefore, the studied prodrugs were effectively delivering their parent drugs into the mouse brain and some of the prodrugs had only a limited premature bioconversion in the periphery (Table 1). However, the aminopeptidase B protein quantification should also be carried out for human tissues, to obtain reliable translation from rodents to humans.

It was also concluded in this study that the aromatic promoieties most likely fit better for aminopeptidase B than the aliphatic promoieties (L-Phe derivatives vs. L-Lys derivatives), but the structural features of the parent drug (the rigidity) most likely define whether the prodrugs are substrates of aminopeptidase B or not (Table 3). Curiously, all the studied parent drugs also contained at least one aromatic ring, but it was not enough to have strong interactions with aminopeptidase B in the case of aliphatic promoieties (prodrugs **3** and **4**). Overall, the aromaticity requirement in the amino acid promoiety seems to be against the original name and substrates of this enzyme, *i.e.*, arginyl aminopeptidase removing arginine and/or lysine residues from the *N*-terminus of peptides. Therefore, more detailed interaction of the compounds within the enzyme catalytic pocket needs to be clarified thoroughly in the future by computational methods.

However, not all the studied aromatic prodrugs were bioconverted by aminopeptidase B, *e.g.*, prodrug **6** (salicylic acid prodrug, the smallest prodrug size-wisely in this series) was not an aminopeptidase B substrate, but was bioconverted in the brain by some other, yet unidentified enzyme(s) (Montaser et al., 2020). It is also highly likely, that the bioconversion of compound **6** is *e.g.*, neurons-specific, and therefore, no bioconversion was seen in astrocytes or microglia cell homogenates (Table 4). It has been reported that aminopeptidase NAP and NAP2 (belonging to the EC 3.4.11 family) are neuron-specific enzymes and can thus, offer a regio-selective bioconversion for some prodrugs (Hui et al., 1998; Hui and Hui, 2008). Nevertheless, it is also possible that other enzymes expressed in the brain are involved in the bioconversion of all the studied prodrugs, in addition to aminopeptidase B. With the non-targeted (global) proteomic approach, we identified several other prodrug bioconverting enzyme candidates from the mouse brain tissue and immortalized microglia. These enzymes include at least exopeptidases, such as aminopeptidase N (ANPEP, EC 3.4.11.2), leucine-cysteinyl aminopeptidase (LNPEP, EC 3.4.11.3), cytosol alanyl aminopeptidase (LAP3, EC 3.4.11.14), methionyl aminopeptidase (METAP1, EC 3.4.11.18), and aspartyl aminopeptidase (DNPEP, EC 3.4.11.21) that can hydrolyze terminal amino acids (Figure 3). From these, leucine-cysteinyl aminopeptidase and methionyl aminopeptidase were expressed in the brain (and liver) but not in plasma. However, several endopeptidases, like cathepsin B, D, S, and Z, were also identified from mouse brain (Figure 3). Although they break peptide bonds of non-terminal amino acids, they may be responsible for prodrug bioconversion. Such an example is cathepsin A or carboxypeptidase Y (CPA, EC 3.4.16.5), which bioconverts tenofovir alafenamide, an antiretroviral agent against human immunodeficiency virus (HIV) (Birkus et al., 2007). However, less is known of other cathepsins as prodrug hydrolyzing enzymes. Notably, an enzyme belonging to the same family with cathepsin A, carboxypeptidase D (CPD, EC 3.4.16.6) was also found selectively expressed in the mouse brain (not expressed in the liver or plasma). In addition, dipeptidyl peptidases 1–3 and tripeptidyl peptidase 2, which all may bear a potential as prodrug bioconverting enzymes, were identified from the mouse brain and not in plasma. At least, dipeptidyl peptidase 4 (DPP4, EC 3.4.14.5) has been studied as a selective prodrug-activating enzyme in cancer cells (Dahan et al., 2014). However, the possibility of all the above-mentioned enzymes being the main bioconverting enzyme of the studied prodrugs was ruled out by the fact that this enzyme was cobalt-dependent and activated in elevated pH (8.5), which is optimal only for aminopeptidase B from the listed enzymes in Figure 3.

Lastly, it is worthwhile to point out that substituting zinc ions with cobalt ions is a very useful tool to explore the catalytic properties of enzymes (Kobayashi and Shimizu, 1999). Cobalt is an essential cofactor in many cellular functions (Kobayashi and Shimizu, 1999; Heffern et al., 2013; Khrustalev et al., 2019), but it can also restore the activity of many enzymes in the absence of zinc ions and even accelerate enzyme activity (Fukasawa et al., 2011). However, when working with cells, attention should be paid to the ingredients in the media, since it has been reported that *e.g.*, DMEM has a higher content of histidine, which can chelate cobalt ions, compared to RPMI 1640 and DMEM/F12 mediums, which seems to be a better choice for cobalt-related studies, in which growing media is required (Torii et al., 2011). It is also hoped that this study encourages researchers to explore the prodrug bioconverting enzymes in more details in the future rather than referring that they were hydrolyzed by ubiquitous hydrolysing enzymes and the prodrugs were stable *in vitro*, but released their parent drug *in vivo*.

## Conclusion

In summary, this study proves the importance of identifying the prodrug bioconverting enzymes, particularly when site-selective prodrug delivery is needed to be achieved. LAT1-utilizing amide prodrugs having aromatic amino acid promoiety and at least one aromatic ring, preferably too aromatic rings in their parent drug with a flexible overall structure, were effectively hydrolyzed by aminopeptidase B in the presence of cobalt ions. Increasing the pH to 8.5 accelerated the bioconversion rate and adding non-specific peptidase inhibitor bestatin inhibited the hydrolysis *via* aminopeptidase B. This enzyme is highly expressed in the mouse brain compared to plasma and liver and particularly in parenchymal cells (microglia), in which LAT1-utilizing compounds are accumulated. However, aminopeptidase B was not bioconverting all the studied prodrugs that were releasing their parent drug *in vivo* in the mouse brain. Therefore, more efforts should be paid to structure activity relationships of amide prodrugs biocoverting enzymes as well as to identifying other possible brain-selective enzymes in the future.

## Data Availability

The data presented in the study are deposited in the ProteomeXchange Consortium via the PRIDE partner repository with the dataset identifier PXD037260.

## Abbreviations

AChE, acetylcholinesterase; BBB, blood-brain barrier; BChE, butyrylcholinesterase; CES, carboxylesterases; CNS, central nervous system; FDA, U.S. Food and Drug Administration; LAT1, L-type amino acid transporter 1; PON, paraoxygenase.
